# Integrating genomics and AI to uncover molecular targets for mRNA vaccine development in lupus nephritis

**DOI:** 10.3389/fimmu.2024.1381445

**Published:** 2024-10-04

**Authors:** Lisha Mou, Ying Lu, Zijing Wu, Zuhui Pu, Meiying Wang

**Affiliations:** ^1^ Department of Rheumatology and Immunology, Institute of Translational Medicine, Health Science Center, The First Affiliated Hospital of Shenzhen University, Shenzhen Second People’s Hospital, Shenzhen, Guangdong, China; ^2^ MetaLife Center, Shenzhen Institute of Translational Medicine, Shenzhen, Guangdong, China; ^3^ Imaging Department, The First Affiliated Hospital of Shenzhen University, Shenzhen Second People’s Hospital, Shenzhen, Guangdong, China

**Keywords:** systemic lupus erythematosus, lupus nephritis, genomics, single cell, single-cell RNA sequencing, non-negative matrix factorization (NMF), machine learning, functional network analysis

## Abstract

Lupus nephritis (LN), a complex complication of systemic lupus erythematosus, requires in-depth cellular and molecular analysis for advanced treatment strategies, including mRNA vaccine development. In this study, we analyzed single-cell RNA sequencing data from 24 LN patients and 10 healthy controls, supplemented by bulk RNA-seq data from additional LN patients and controls. By applying non-negative matrix factorization (NMF), we identified four distinct leukocyte meta-programs in LN, highlighting diverse immune functions and potential mRNA vaccine targets. Utilizing 12 machine learning algorithms, we developed 417 predictive models incorporating gene sets linked to key biological pathways, such as MTOR signaling, autophagy, Toll-like receptor, and adaptive immunity pathways. These models were instrumental in identifying potential targets for mRNA vaccine development. Our functional network analysis further revealed intricate gene interactions, providing novel insights into the molecular basis of LN. Additionally, we validated the mRNA expression levels of potential vaccine targets across multiple cohorts and correlated them with clinical parameters such as the glomerular filtration rate (GFR) and pathological stage. This study represents a significant advance in LN research by merging single-cell genomics with the precision of NMF and machine learning, broadening our understanding of LN at the cellular and molecular levels. More importantly, our findings shed light on the development of targeted mRNA vaccines, offering new possibilities for diagnostics and therapeutics for this complex autoimmune disease.

## Introduction

Lupus nephritis (LN), a complex and severe manifestation of systemic lupus erythematosus, presents significant challenges in both diagnosis and treatment (1). The heterogeneous nature and intricate pathophysiology of LN call for advanced and nuanced research approaches (2). Although traditional methodologies have provided valuable insights, there is a growing need for more advanced techniques to fully understand and address the complexities of LN.

In this study, we selected four specific gene groups (MTOR-related genes, autophagy-related genes, Toll-like receptor-related genes, and adaptive immune system-related genes) based on their critical roles in key biological processes and pathways relevant to LN pathogenesis. The mTOR signaling pathway is vital for cellular metabolism and immune function, autophagy maintains cellular homeostasis and modulates immune responses, Toll-like receptors are central to innate immunity and inflammation, and the adaptive immune system is crucial for immune regulation and autoimmunity. By focusing on these pathways, we aimed to uncover the multifaceted mechanisms underlying LN and identify potential therapeutic targets.

The advent of single-cell RNA sequencing (scRNA-seq) technologies has revolutionized our understanding of diseases (3–5). These technologies provide detailed insights into the cellular mechanisms underlying systemic lupus erythematosus and LN (6, 7), revealing the diverse cell populations and unique expression landscapes critical to the disease pathogenesis. By dissecting these heterogeneous cell populations, scRNA-seq enables the identification of novel cell types and states that are instrumental in driving LN pathology.

Complementing scRNA-seq, machine learning algorithms (8–10) have emerged as powerful tools for analyzing the vast and complex datasets characteristic of modern genomics. In the context of LN, these algorithms synthesize and interpret large-scale genomic data, facilitating the development of predictive models that uncover underlying patterns and correlations. Such models are crucial for identifying potential biomarkers and novel therapeutic targets, particularly in conditions where traditional statistical methods are limited.

Recent advances in scRNA-seq combined with bulk RNA-seq and machine learning have significantly enhanced our understanding of complex diseases such as LN. Previous studies have demonstrated the utility of integrating these technologies to uncover disease mechanisms and develop predictive models. For instance, researchers have successfully applied these methods in various contexts, including LN and other diseases (11–15). These studies have shown that combining scRNA-seq with bulk RNA-seq provides a comprehensive view of cellular and molecular dynamics, enabling the identification of novel biomarkers and therapeutic targets.

Building on these foundational works, our study aims to further advance the field by employing non-negative matrix factorization (NMF) (16, 17) and a diverse array of machine learning algorithms to dissect the genetic and cellular complexities of LN. We aimed to perform a comprehensive cellular and molecular analysis of LN utilizing NMF to dissect its genetic and cellular intricacies. Our approach not only enhances the understanding of LN but also sets the stage for the development of novel therapeutic strategies, including the potential design of mRNA vaccines.

In the context of mRNA vaccine development, our research has additional significance. The identification and characterization of key antigens through our analysis could provide the basis for designing mRNA vaccines tailored to the LN. These vaccines, which target specific antigens identified in our study, could help modulate the immune response in LN patients, potentially offering a new approach for treatment. This aligns with the growing interest in personalized medicine and the need for treatments that address the unique aspects of autoimmune diseases such as LN.

Our use of state-of-the-art technologies to unravel the cellular and molecular complexities of LN aims not only to deepen our understanding of the disease but also to explore innovative treatment options, such as mRNA vaccines. By integrating scRNA-seq for cellular profiling, NMF for pattern identification, and machine learning for predictive modeling, we sought to elucidate the intricate genetic and cellular interactions in LN. This comprehensive approach has the potential to transform the management of LN, facilitating the transition to targeted treatments and a move toward more personalized medical interventions.

## Materials and methods

### Data acquisition and preprocessing for mRNA vaccine target identification

In our study, we obtained single-cell RNA sequencing (scRNA-seq) data for twenty-four patients with lupus nephritis (LN) and ten healthy controls from a previous study (18). These high-resolution data were crucial for investigating cellular heterogeneity in LN, with a specific focus on identifying potential mRNA vaccine targets. Additionally, we integrated bulk RNA sequencing (RNA-seq) datasets from the Gene Expression Omnibus (GEO) database to construct a more comprehensive patient cohort for the construction of our machine learning models. Rigorous preprocessing, including normalization, batch effect correction, and quality control, was applied to ensure data quality and comparability, which are essential for accurate target identification. Details for the four bulk RNA-seq cohorts are shown in **Supplementary Table 1** (19–22).

### Gene set curation for discovery of mRNA vaccine targets

To identify potential targets for mRNA vaccine development, we curated four gene sets associated with critical signaling pathways, including the MTOR, autophagy, Toll-like receptor, and adaptive immune system pathways. These curated gene sets represent a spectrum of biological functions and processes pivotal for LN, and their analysis was integral to our approach to identifying mRNA vaccine targets. The details for the four gene sets are as follows:

1. MTOR-related genes (MTORGs): Sourced from MSigDB, these genes are involved in the mTOR signaling pathway, which is crucial for cell growth, proliferation, motility, survival, protein synthesis, and transcription.
2. Autophagy-related genes (AutRG): Collected from a range of databases, including HADb, AUTOPHAGY DATABASE, and MSigDB, as well as recent scientific publications, these genes are essential in the process of autophagy, the cellular mechanism of removing damaged cells to regenerate newer, healthier cells.
3. Toll-like receptor-related genes (TolRGs): Based on recent scientific studies, these genes are crucial for the Toll-like receptor (TLR) signaling pathway, which is known for its role in the innate immune system.
4. Adaptive immune system-related genes (AISRGs): Collected from Reactome, these genes are crucial for the adaptive immune response, offering insights into the host-specific immune defense mechanisms against pathogens.

These curated gene sets, which represent a broad spectrum of biological functions and processes, establish a foundation for our comprehensive analyses. By focusing on these specific pathways, we sought to elucidate the multifaceted nature of LN at the molecular level, aiming to elucidate the genetic framework and regulatory networks pivotal for LN.

### Single-cell data analysis for vaccine target identification

Our single-cell data analysis began with a detailed examination of samples from 24 LN patients and 10 healthy controls. Using the Seurat package (version 4.4.0) (23), a critical tool for single-cell genomics, we performed initial data filtering to ensure the quality and integrity of our analysis. The process involved the use of rigorous quality control measures to filter out low-quality cells and normalize the data for downstream analysis. For specific quality control metrics and annotation procedures, we followed the guidelines outlined in the original article.

We employed a two-step approach for dimensionality reduction and visualization to elucidate the cellular landscape of LN. Initially, we utilized principal component analysis (PCA) to reduce the high-dimensional scRNA-seq data to a lower-dimensional space. PCA is a robust and widely used method that highlights the primary sources of variance in a dataset. This initial step is critical for data refinement and identifying the major patterns, which facilitates subsequent clustering analyses. Following PCA, we applied t-distributed stochastic neighbor embedding (t-SNE) for further dimensionality reduction and visualization. t-SNE is particularly effective in capturing complex, nonlinear relationships within the data and preserving local structures, making it well suited for visualizing distinct cellular clusters. The combination of PCA and t-SNE enabled us to achieve a detailed and interpretable representation of the cellular heterogeneity in LN.

The essence of our single-cell analysis was the identification and characterization of distinct cellular clusters within the LN and control cohorts. With the use of Seurat clustering algorithms, we were able to delineate these clusters based on their unique gene expression profiles. This approach allowed us to segregate the cell populations into discernible groups, thereby enabling a more granular understanding of the cellular composition of LN. Postclustering, our analysis characterized four major cell types predominant in the LN microenvironment: T/NK cells, myeloid cells, B cells, and epithelial cells. This categorization was based on the expression of canonical cell type-specific markers. Furthermore, we identified 22 subcell types within these major categories, each representing unique functional states and potential roles in the pathophysiology of LN. We focused on identifying and characterizing distinct cellular clusters within the LN and control cohorts, with an emphasis on finding unique functional states and potential roles in LN pathophysiology that could inform mRNA vaccine development.

### Application of non-negative matrix factorization (NMF) in identifying vaccine targets

We employed the NMF algorithm to decompose the high-dimensional scRNA-seq data into a set of basic components and corresponding coefficients. This method is particularly effective for uncovering underlying structures in complex biological data, such as transcriptional programs active in different cell types. Specifically, we applied NMF to analyze leukocyte gene expression in LN patients, which is crucial for identifying nonoverlapping gene modules that could serve as targets for mRNA vaccine development.

Our NMF application followed these steps:

1. Standardization: Negative values in the data were standardized to zero to ensure compatibility with the NMF algorithm.
2. Algorithm Execution: We used the consensus NMF (cNMF) algorithm (17) and ran it for 100 iterations with the number of components (k) ranging from 4 to 9. The optimal number of components was determined using diagnostic plots, as recommended in the cNMF tutorial (https://github.com/dylkot/cNMF).
3. Identification of Meta-Programs: The cNMF algorithm identified four distinct leukocyte meta-programs (MP1-MP4), each representing a unique transcriptional signature. These meta-programs were characterized by clusters of top-scoring genes, indicating their prominence in the LN transcriptional landscape.

A critical step in our NMF application was the identification of nonoverlapping gene modules, which was achieved through a novel gene ranking algorithm. This algorithm involved constructing two distinct ranking matrices. The first matrix detailed how each gene contributed to the different factors, while the second matrix ranked the factors based on their contribution to each gene. Genes were incrementally assigned to each factor based on their contribution levels until a gene’s contribution to another factor became more significant, as indicated by a change in their rank across factors.

To further dissect the expression patterns, we employed Pearson correlation analysis coupled with hierarchical clustering. This approach allowed us to delve more deeply into the relationships and similarities between different gene expression programs. The culmination of this intricate analytical process was the identification of four distinct meta-programs within the leukocytes of LN patients. These clusters, identified as meta-program 1 to 4, comprise the top-ranking genes, each signaling a distinct transcriptional signature within the landscape of LN. These meta-programs represent unique and coherent gene expression patterns, shedding light on the underlying biological processes and pathways active in LN.

### Development of a predictive model for LN status and vaccine targeting

We developed predictive models by examining the intersection of meta-program 1 with key gene sets (MTORGs, AutRGs, TolRGs, and AISRGs), focusing on identifying gene interactions and expression patterns critical for LN and potential vaccine targets. We employed twelve different machine learning algorithms, including LASSO (24), Ridge (24), Elastic network (24), Stepglm (25), SVM (26), GlmBoost (27), LDA (28), plsRglm (29), RSF (30), GBMs (31), XGBoost (32), and naive Bayes (25). These algorithms were implemented using the R programming language and associated packages. Detailed descriptions of each algorithm, including the parameter settings and configurations, are provided in the **Supplementary Materials** (**Supplementary Table 2**). The R scripts are available in a public GitHub repository (https://github.com/lishamou/LN_ML/).

This diverse toolkit was essential for prioritizing diagnostic accuracy and emphasizing key genes involved in LN pathogenesis. This diverse toolkit included the choice of these algorithms because of their proven efficacy in various predictive modeling scenarios, especially in biomedical applications. We constructed a total of 417 predictive models, each representing a unique combination of gene sets and algorithmic configurations. These models were optimized for efficiency, prioritizing diagnostic accuracy and emphasizing the importance of key genes in LN pathogenesis. The concordance index (C-index) was used to assess the models’ predictive performance, providing a clear measure of their diagnostic power.

The initial phase of model development utilized a combined training dataset from the GSE32591 and GSE113342 cohorts, providing a rich and diverse foundation for preliminary model training. Model validation was subsequently conducted using the GSE200306 and GSE81622 cohorts. These additional cohorts ensured the robustness and generalizability of our models across different patient populations and sample types. This step was critical for assessing the models’ performance consistency and applicability across different patient groups. Our performance assessment focused on the C-index, a widely recognized metric for evaluating predictive accuracy. By calculating the C-index for each model across all cohorts, we achieved a comprehensive view of the discriminative ability of the models. This rigorous validation process confirmed the reliability and potential clinical applicability of the models, demonstrating their effectiveness in diverse sample sets and laying the groundwork for their future integration into clinical practice for LN.

### Expression validation of potential mRNA vaccine targets in an in-house cohort

Using q−PCR, we validated the expression of potential mRNA vaccine targets within our in-house cohort. Blood samples were collected from both healthy controls and LN patients at Shenzhen Second People’s Hospital. All participants provided written informed consent, and the study received ethical approval (Approval No. 20220824001). RNA extraction, reverse transcription, and q-PCR were carried out according to previously established protocols. Samples from LN patients (n=3) and healthy volunteers (n=3) were analyzed. The specific sequences of primers used in the analysis are listed in **Supplementary Table 3**.

### Expression validation and clinical correlations of potential mRNA vaccine targets

The expression levels of potential mRNA vaccine targets and clincal correlations were examined using five datasets (20, 33–35) (**Supplementary Table 4**). To understand the clinical significance of these targets, we analyzed their correlation with critical clinical parameters, including the glomerular filtration rate (GFR) and pathological stage.

### Functional network analysis for discovery of mRNA vaccine targets

To elucidate the complex interplay among key gene sets in LN, we used the GeneMANIA database (http://genemania.org/) (36, 37) to construct comprehensive functional networks. This analysis included hub genes from four critical sets: MTORGs, AutRGs, TolRGs, and AISRGs. We carefully mapped the interactions within and between these gene sets, considering various interaction types, such as coexpression, physical interactions, colocalization, shared pathways, shared protein domains, and predicted and genetic interactions.

In addition to network construction, the GeneMania results were used to perform Gene Ontology (GO) and KEGG enrichment analysis for each of these hub gene sets. We augmented our analysis using Cytoscape software (version 3.10.1) (38), an advanced platform adept at visualizing intricate networks and assimilating various biological datasets. This network mapping and GO and KEGG enrichment analysis were vital for understanding how these hub genes contribute to LN and for guiding mRNA vaccine target discovery.

To effectively communicate the results of our GO enrichment analysis, we utilized the UpSet diagram. This visualization tool provided a clear and concise representation of the overlapping and unique GO terms across the hub gene sets. The diagram enabled us to illustrate the convergence of biological processes and functions among the MTORGs, AutRGs, TolRGs, and AISRGs, thereby offering a comprehensive view of the multifaceted roles these genes play in LN. Through this functional network analysis, we aimed not only to map the intricate gene–gene interactions but also to interpret the broader biological implications of these interactions. The combined approach of network mapping and GO enrichment analysis provided us with a deeper understanding of how these hub genes contribute to the pathophysiology of LN, potentially guiding future therapeutic strategies and biomarker discovery.

### Mechanistic diagram drawing

We utilized Adobe Illustrator to visually represent the complex molecular interactions and pathways implicated in LN.

### Statistical analysis

Statistical analyses of both single-cell and bulk RNA sequencing datasets were conducted using R (version 4.3.1), with a stringent significance threshold set at a P value of less than 0.05. For the predictive models, we used the concordance index (C-index) to assess the predictive accuracy, providing a framework for evaluating the performance of our models in identifying potential mRNA vaccine targets.

## Results

### Cellular diversity in patients with lupus nephritis revealed by single-cell RNA sequencing analysis

**Figure 1:**
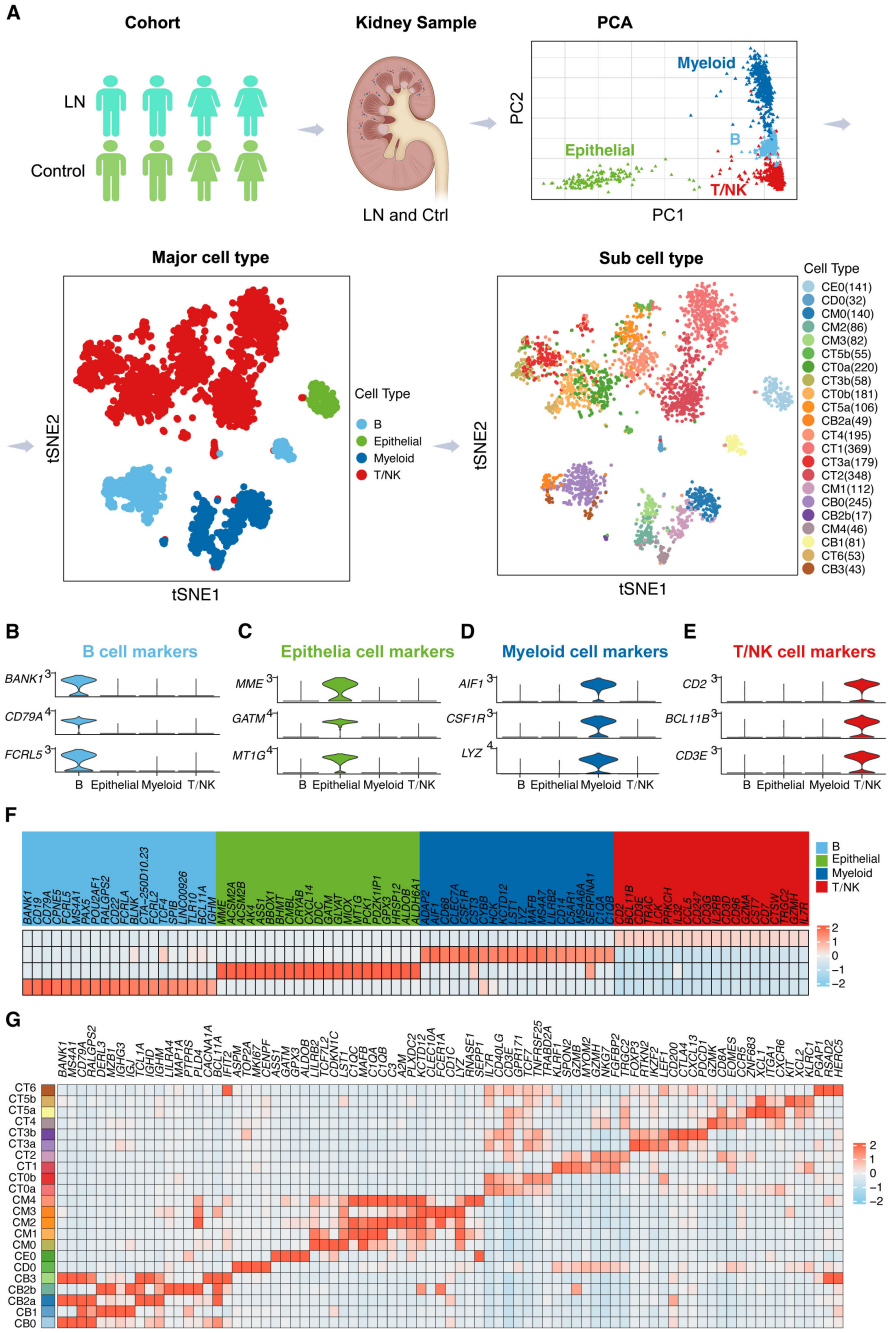
Dissecting the Single-Cell Landscape **(A)** Workflow of single-cell RNA sequencing (scRNA-seq) analysis for lupus nephritis (LN) patients. We analyzed scRNA-seq data from kidney samples from 24 LN patients and 10 healthy controls. Principal component analysis (PCA) plot delineating four principal cell types in the scRNA-seq dataset. Furthermore, the t-SNE plots provide a spatial representation of four principal cell types and 22 subcell types in the LN landscape. Violin plots illustrating marker gene expression patterns across identified major cell types, including **(B)** B cells, **(C)** epithelial cells, **(D)** myeloid cells, and **(E)** T/NK cells. **(F)** A heatmap showing the relative abundance of marker genes across the four major cell types. **(G)** Heatmap showing the relative abundance of marker genes across the 22 subcell types.

In our comprehensive analysis of LN, we utilized single-cell RNA sequencing (scRNA-seq) to investigate kidney samples from 24 LN patients and 10 healthy controls. The workflow for scRNA-seq analysis is depicted in **Figure 1A**. The principal component analysis (PCA) plot identifies four major cell types present in the scRNA-seq dataset: B cells, epithelial cells, myeloid cells, and T/NK cells. This dimensionality reduction technique provides an initial overview of the cellular diversity within the samples.

Subsequently, t-distributed stochastic neighbor embedding (t-SNE) plots were generated to provide a spatial representation of these four principal cell types, further distinguishing 22 subcell types within the LN landscape. The t-SNE visualization enables a clearer understanding of the cellular heterogeneity in LN, highlighting the distribution and relationships among different cell populations.

In **Figures 1B–E**, violin plots depict the distribution and variability of marker gene expression within the major cell types, providing insights into the density and range of expression levels. Specifically, **Figure 1B** focuses on B cells, **Figure 1C** on epithelial cells, **Figure 1D** on myeloid cells, and **Figure 1E** on T/NK cells. Conversely, t-SNE plots were generated to illustrate the spatial distribution and clustering of these marker genes across four major cell types, highlighting the relationships and proximities between different cellular subpopulations (**Supplementary Figure 1**). This combination of visualizations offers a comprehensive view of the cellular landscape in the LN, integrating statistical distribution with spatial organization.

Furthermore, the heatmap in **Figure 1F** shows the relative abundance of marker genes across the four major cell types, providing a quantitative view of gene expression levels. This visualization underscores the heterogeneity and complexity of gene expression patterns within the major cell types.

In addition, the detailed heatmap in **Figure 1G** illustrates the relative abundance of marker genes across the 22 subcell types identified in the t-SNE analysis. This detailed further elucidates the specific gene expression signatures associated with each subcell type, offering deeper insights into the cellular and molecular landscape of LN.

### Expansive development of predictive models to illuminate key genomic associations in LN for mRNA vaccine development

**Figure 2:**
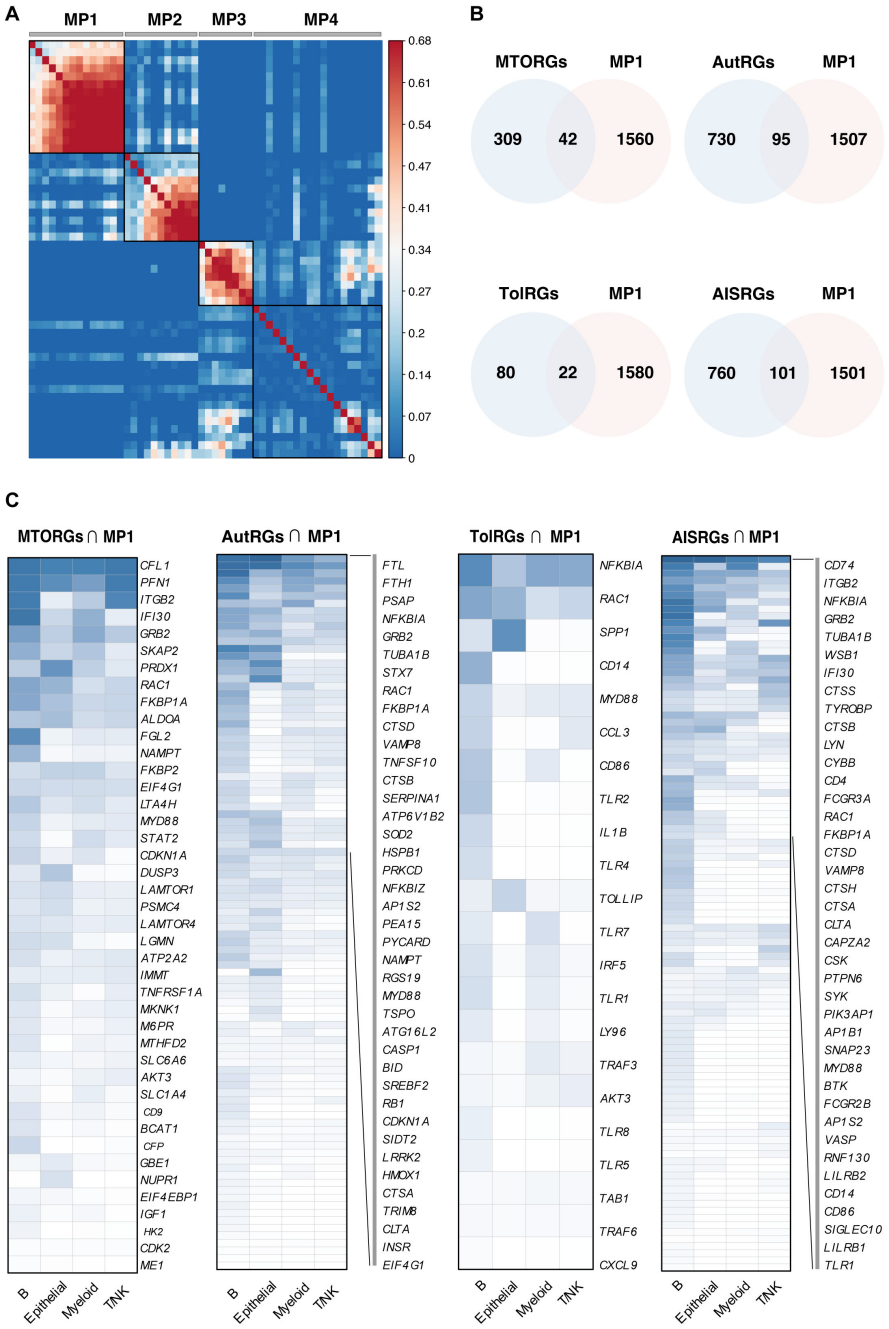
Leukocyte Meta-Programs and Identification of Key Pathway-Related Genes. **(A)** Identification of four distinct leukocyte meta-programs (MP1-MP4) from the scRNA-seq dataset, each with a unique transcriptional signature, which could guide the development of targeted mRNA vaccines. **(B)** Four Venn diagrams showing the intersections among four gene sets related to critical pathways identified with MP1, indicating potential molecular targets for mRNA vaccines. **(B)** Heatmap providing a comparative view of gene expression levels within the major cell types of the LN single-cell dataset, demonstrating the differential expression and potential functional roles of these genes in vaccine target selection.

A novel aspect of our study was the use of consensus non-negative matrix factorization (cNMF) to analyze leukocytes from LN patients, leading to the identification of four distinct transcriptional meta-programs, as shown in **Figure 2A**. These meta-programs, labeled 1 to 4, were characterized by gene clusters with high expression levels, indicating their crucial roles in the LN transcriptome. Each meta-program represented a unique transcriptional profile, enhancing our understanding of leukocyte gene expression in LN. The diversity of these meta-programs revealed complex gene interactions and suggested that various immunological pathways and cellular activation states are intrinsic to LN.

These findings are particularly significant for the development of mRNA vaccines targeting the LN. The identified cell types and transcriptional profiles provide a rich source of potential antigens for vaccine development. In particular, the unique expression patterns of B cells and T/NK cells, key players in the immune response, offer promising targets for vaccine design. Our focus on elucidating these meta-programs and cellular diversity aims to facilitate the identification of specific antigens that could be utilized in mRNA vaccines to modulate the immune response in LN patients.

By charting these expression profiles, we have established a foundation for further investigations into immune cell behavior in LN. This detailed understanding of the genetic and cellular makeup of LN is crucial for directing the development of future predictive models, which could be instrumental in identifying suitable antigen targets for mRNA vaccine development. Our results not only advance the knowledge of LN at the genetic level but also contribute to the emerging field of personalized vaccine therapy, potentially transforming the approach to this complex autoimmune disease.

To revolutionize the diagnostic and therapeutic landscape for LN, we developed an extensive suite of 417 predictive models, integrating data from the first of our four identified meta-programs with key gene sets that encompass vital biological pathways. These pathways, from MTOR signaling to adaptive immunity, are critical to the pathophysiology of LN. Our comprehensive gene list included 42 MTOR-related genes (MTORGs), 95 autophagy-related genes (AutRGs), 22 Toll-like receptor-related genes (TolRGs), and 101 adaptive immune system-related genes (AISRGs), each revealing distinct expression patterns crucial for understanding LN (**Figure 2B**). The number of models developed for each gene group (MTORGs, AutRGs, TolRGs, and AISRGs) was influenced by the combination of various machine learning algorithms and their parameter settings. Initially, all possible models were generated using different algorithmic configurations. During the evaluation process, models with suboptimal performance, such as those with low concordance index (C-index) values, were excluded from the final analysis. For instance, we developed 105 models for MTORGs, 101 models for AutRGs, 110 models for TolRGs, and 101 models for AISRGs. This comprehensive approach ensured that only the most robust and accurate models were retained, allowing for a thorough evaluation of the predictive capabilities of each gene group in the context of LN. The detailed lists of all genes included in the MTOG, AutRG, TolRG, and AISRG groups, along with their annotations, are provided in **Supplementary Table 5**. The heatmap in **Figure 2C** encapsulates these expression patterns across the gene sets, providing a visual guide to the intricate genomic associations in LN.

**Figure 3:**
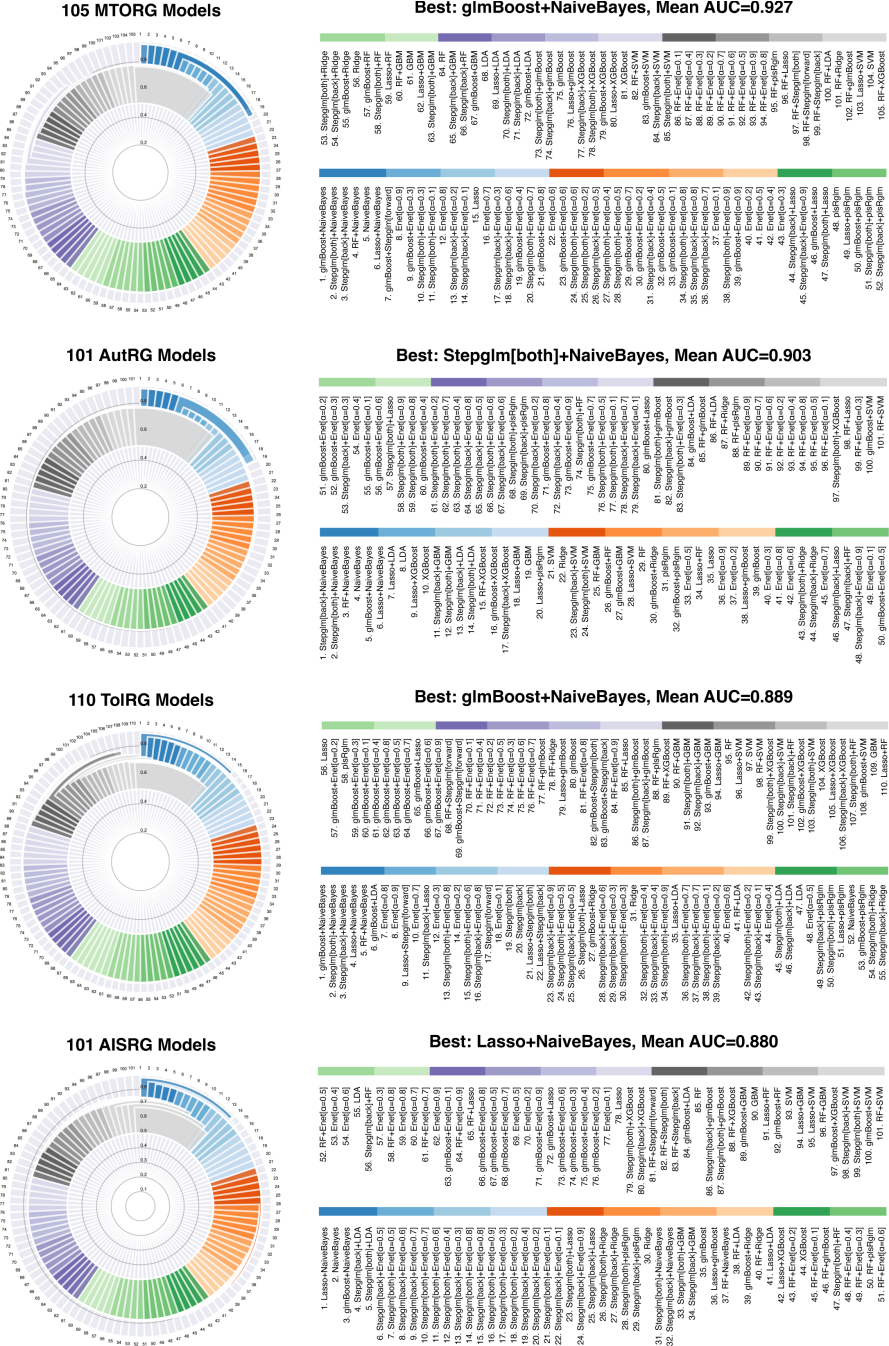
Assessment of Predictive Model Performance in Identifying mRNA Vaccine Targets. This composite figure presents the mean concordance index (C-index) results for our suite of machine learning models, stratified by gene set and algorithm combination. The subpanels detail the performance of the models corresponding to each gene set (MTORG, AutRG, TolRG, and AISRG) in the training cohorts, confirming the diagnostic accuracy of our models in identifying mRNA vaccine targets.

Utilizing a diverse array of 12 machine learning algorithms, we assessed the diagnostic accuracy of our models using the C-index. The models demonstrated high predictive accuracy, with significant results across our training and validation datasets, as detailed in **Figure 3**, **Supplementary Figures 2**-**4**, and **Supplementary Tables 6**-**9**.

Model development commenced with a training dataset amalgamating data from cohorts GSE32591 and GSE113342. Model validation was conducted with two additional cohorts, GSE200306 and GSE81622, a crucial step for verifying the models’ consistent performance and generalizability across diverse patient populations.

Notably, models utilizing MTORGs with combinations of glmBoost plus naive Bayes algorithms and AutRGs with a combination of Stepglm [bothward] plus naive Bayes demonstrated exceptional predictive strength, achieving mean AUC values of 0.927 and 0.903 across both training and validation datasets, respectively. These results suggest their potential for identifying key targets for mRNA vaccine development (**Figure 3**). The performance of blood sample analyses (GSE81622) was particularly noteworthy, suggesting a less invasive approach for LN diagnostics and providing a potential pathway for identifying blood-based biomarkers for vaccine development (**Supplementary Figure 4**).

By exploring the expression of hub genes within single-cell datasets, we discovered notable expression patterns in myeloid cells (**Supplementary Figure 5**). These insights are invaluable for mRNA vaccine development, as they highlight key genes that could be targeted to modulate the immune response in LN patients.

### Deciphering LN mechanisms: informing mRNA vaccine development through interaction networks, GO, and KEGG enrichments

**Figure 4:**
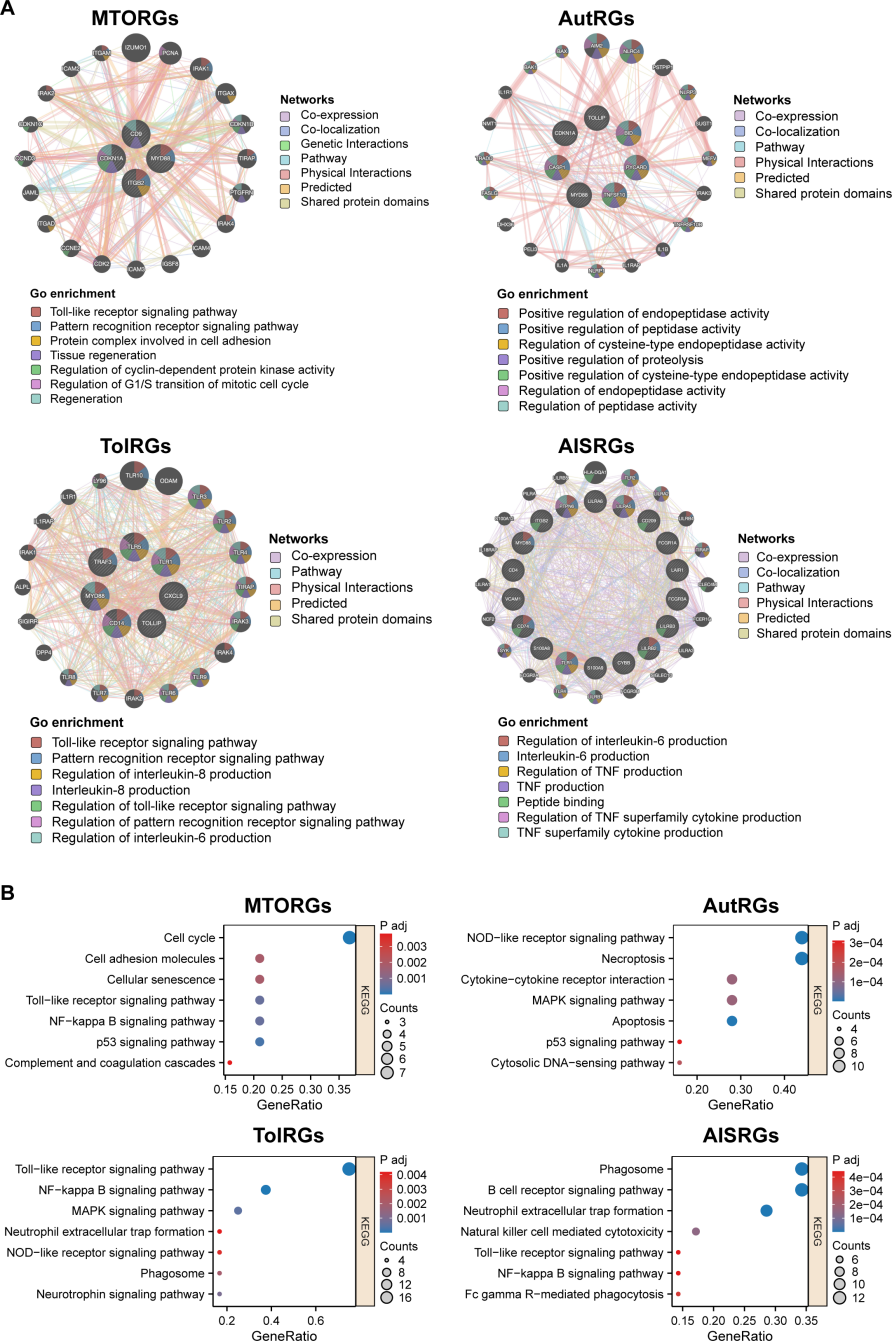
Exploring the Gene Interaction Network for mRNA Vaccine Target Discovery in LN Pathogenesis. **(A)** GeneMANIA network analysis revealed a comprehensive array of interactions among the hub genes relevant to LN, including MTORG, AutRG, TolRG, and AISRG. The types of interactions (co-expression, physical contacts, co-localization, pathway sharing, and predicted genetic links) are denoted by colored lines. Nodes within the network are color-coded according to enriched Gene Ontology (GO) terms, providing insights into the functions of these genes and their interconnectedness, which is crucial for identifying potential targets for mRNA vaccine development. **(B)** KEGG pathway enrichment analysis.

To further inform our mRNA vaccine development strategy, we conducted a comprehensive network analysis using the GeneMANIA database, revealing a complex matrix of gene interactions central to LN pathophysiology (**Figure 4A**). This analysis highlighted the dynamic interplay among coexpressed genes, physical and predicted interactions, shared pathways, and genetic links, emphasizing the diverse biological processes involved in LN.

Significant interactions involving MTORGs, such as *CD9*, *CDKN1A*, *ITGB2*, and *MYD88*, and their interactions with other genes, which form a complex network of immune responses in the LN, were detected. These interactions span a range of biological functions, from Toll-like receptor signaling to cell cycle regulation, all of which are pivotal in understanding and targeting LN.

Our Gene Ontology (GO) enrichment analysis highlighted significant overlaps in key biological processes such as interleukin production and Toll-like receptor signaling (**Figure 4A**, **Supplementary Tables 10**-**13**). The UpSet diagram (**Supplementary Figure 6**, **Supplementary Table 14**) visually illustrates these functional enrichments, clarifying the shared biological significance of these pathways in LN.

Our study utilized KEGG pathway enrichment analysis to explore the functional implications of the protein-protein interactions among various groups of hub genes identified in LN using GeneMania results (**Figure 4B**). The analysis revealed significant enrichment of specific pathways associated with each gene group, providing deeper insights into their roles in LN pathogenesis:

The genes associated with MTORGs were predominantly enriched in pathways that regulate cell proliferation, apoptosis, and immune response, including: (1) Cell Cycle; (2) p53 Signaling Pathway: Critical for apoptosis and cell cycle control, (3) NF-kappa B Signaling Pathway: Key regulator of immune response and inflammation; (4) Toll-like Receptor Signaling Pathway: Important in innate immunity; (5) Cellular Senescence; (6) Cell Adhesion Molecules; (7) Complement and Coagulation Cascades: Essential in inflammation and immune defenses.

TolRGs showed enrichment in pathways involved in immune recognition and response, which are crucial for the activation of adaptive and innate immune systems: (1) Toll-like Receptor Signaling Pathway; (2) NF-kappa B Signaling Pathway; (3) MAPK Signaling Pathway: Involved in cellular proliferation, differentiation, and migration; (4) Neurotrophin Signaling Pathway; (5) Phagosome: Key in pathogen elimination; (6) NOD-like Receptor Signaling Pathway; (7) Neutrophil Extracellular Trap Formation: Important for trapping pathogens.

AutRGs were enriched in pathways that deal with cell death, stress responses, and inflammation: (1) Necroptosis: A form of programmed cell death. (2) NOD-like Receptor Signaling Pathway; (3) Apoptosis (4) MAPK Signaling Pathway (5) Cytokine-cytokine Receptor Interaction; (6) Cytosolic DNA-sensing Pathway; (7) p53 Signaling Pathway.

AISRGs were found to be enriched in pathways that mediate immune cell functions and responses, highlighting their roles in the immune surveillance and response mechanisms: (1) B Cell Receptor Signaling Pathway; (2) Phagosome (3) Neutrophil Extracellular Trap Formation; (4) Natural Killer Cell Mediated Cytotoxicity: (5) Fc gamma R-mediated Phagocytosis; (6) NF-kappa B Signaling Pathway; (7) Toll-like Receptor Signaling Pathway.

### Validation of mRNA expression for potential mRNA vaccine targets

Given the remarkable predictive strength of models utilizing MTORGs with combinations of glmBoost plus naive Bayes algorithms (constructed by *CD9, CDKN1A, ITGB2*, and *MYD88*) and AutRGs with a combination of Stepglm [bothward] plus naive Bayes (constructed by *BID, CASP1, CDKN1A, MYD88, PYCARD, TNFSF10*, and *TOLLIP*), which achieved mean AUC values of 0.927 and 0.903, respectively, we conducted further analyses to study the genes incorporated in these two predictive models. The genes analyzed included *ITGB2, MYD88, CASP1, BID, CDKN1A, PYCARD*, and *TNFSF10* across five cohorts. Two additional genes, *TOLLIP* and *CD9*, were not detected in these cohorts and were therefore excluded from further analysis.

**Figure 5:**
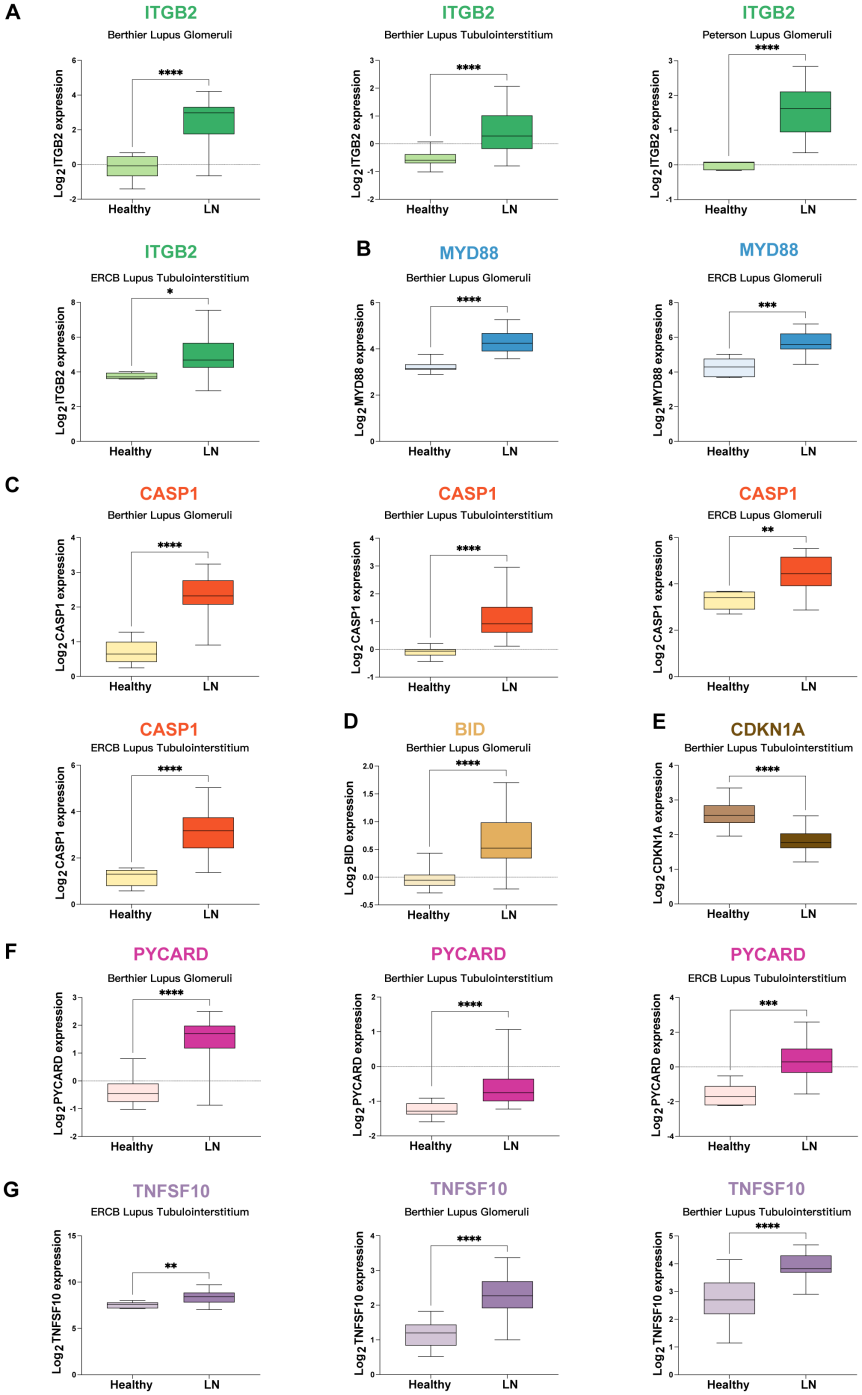
Validation of mRNA Expression for Potential mRNA Vaccine Targets **(A)** Expression levels of *ITGB2* across various cohorts: Berthier Lupus Glomeruli, Berthier Lupus Tubulointerstitium, Peterson Lupus Glomeruli, and the ERCB Lupus Tubulointerstitium. **(B)** *MYD88* expression in the Berthier Lupus Glomeruli and ERCB Lupus Glomeruli cohorts. **(C)** *CASP1* expression in the Berthier Lupus Glomeruli, Berthier Lupus Tubulointerstitium, ERCB Lupus Glomeruli, and ERCB Lupus Tubulointerstitium cohorts. **(D)** *BID* expression in the Berthier Lupus glomeruli cohort. **(E)** *CDKN1A* expression in the Berthier Lupus Tubulointerstitium cohort. **(F)** *PYCARD* expression in the Berthier Lupus Glomeruli, Berthier Lupus Tubulointerstitium, and ERCB Lupus Tubulointerstitium cohorts. **(G)** *TNFSF10* expression in the ERCB Lupus Tubulointerstitium, Berthier Lupus Glomeruli, and Berthier Lupus Tubulointerstitium cohorts. * p < 0.05, ** p < 0.01, *** p < 0.001, **** p < 0.0001.

The mRNA expression levels of potential mRNA vaccine targets were validated across various cohorts: (1) *ITGB2*: Expression levels were assessed in the Berthier Lupus Glomeruli, Berthier Lupus Tubulointerstitium, Peterson Lupus Glomeruli, and ERCB Lupus Tubulointerstitium cohorts (**Figure 5A**). (2) *MYD88*: Expression was analyzed in the Berthier Lupus Glomeruli and ERCB Lupus Glomeruli cohorts (**Figure 5B**). (3) *CASP1*: Expression levels were validated in the Berthier Lupus Glomeruli, Berthier Lupus Tubulointerstitium, ERCB Lupus Glomeruli, and ERCB Lupus Tubulointerstitium cohorts (**Figure 5C**). (4) *BID*: Expression was assessed in the Berthier Lupus Glomeruli cohort (**Figure 5D**). (5) *CDKN1A*: Expression levels were analyzed in the Berthier Lupus Tubulointerstitium cohort (**Figure 5E**). (6) *PYCARD*: Expression was validated in the Berthier Lupus Glomeruli, Berthier Lupus Tubulointerstitium, and ERCB Lupus Tubulointerstitium cohorts (**Figure 5F**). (7) *TNFSF10*: Expression levels were assessed in the ERCB Lupus Tubulointerstitium, Berthier Lupus Glomeruli, and Berthier Lupus Tubulointerstitium cohorts (**Figure 5G**). All of the above genes were upregulated in LN patients, except for *CDKN1A*. Further study focused on the upregulated genes.

### Correlation analysis of potential mRNA vaccine targets with renal function

**Figure 6:**
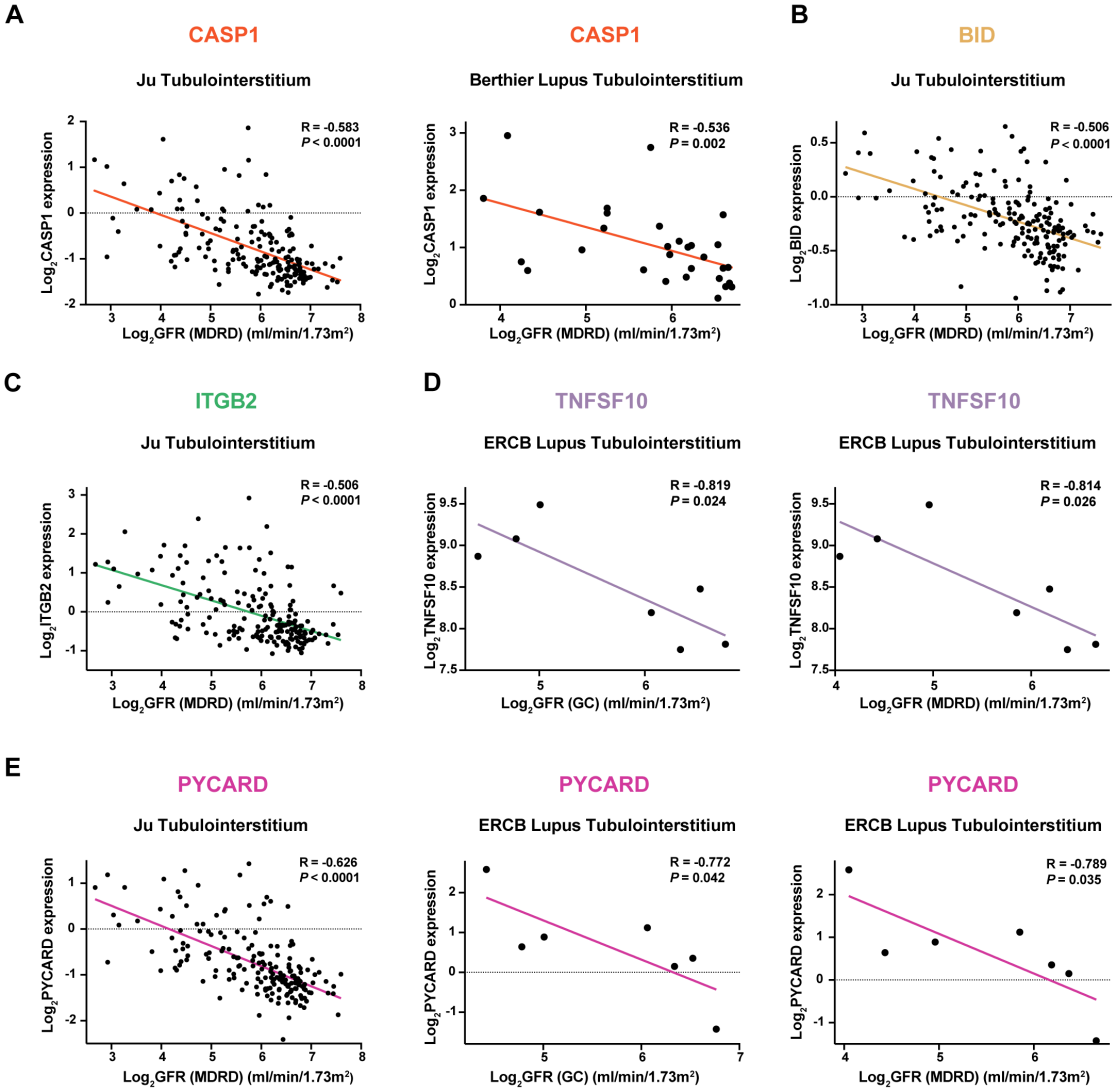
Correlation Analysis of Potential mRNA Vaccine Targets with Renal Function in Three External Validation Cohorts Negative correlation of the following genes with the glomerular filtration rate (GFR): **(A)** *CASP1*, **(B)** *BID*, **(C)** *ITGB2*, **(D)** *TNFSF10*, and **(E)** *PYCARD*.

The correlation of potential mRNA vaccine targets with renal function was investigated by analyzing their association with the glomerular filtration rate (GFR) in three external validation cohorts: (1) *CASP1* showed a negative correlation with the GFR (**Figure 6A**). (2) *BID* exhibited a negative correlation with the GFR (**Figure 6B**). (3) *ITGB2* was negatively correlated with the GFR (**Figure 6C**). (4) *TNFSF10*: *TNFSF10* was negatively correlated with the GFR (**Figure 6D**). (5) The *PYCARD* showed a negative correlation with the GFR (**Figure 6E**).

### Correlation analysis of potential mRNA vaccine targets with pathological stage

**Figure 7:**
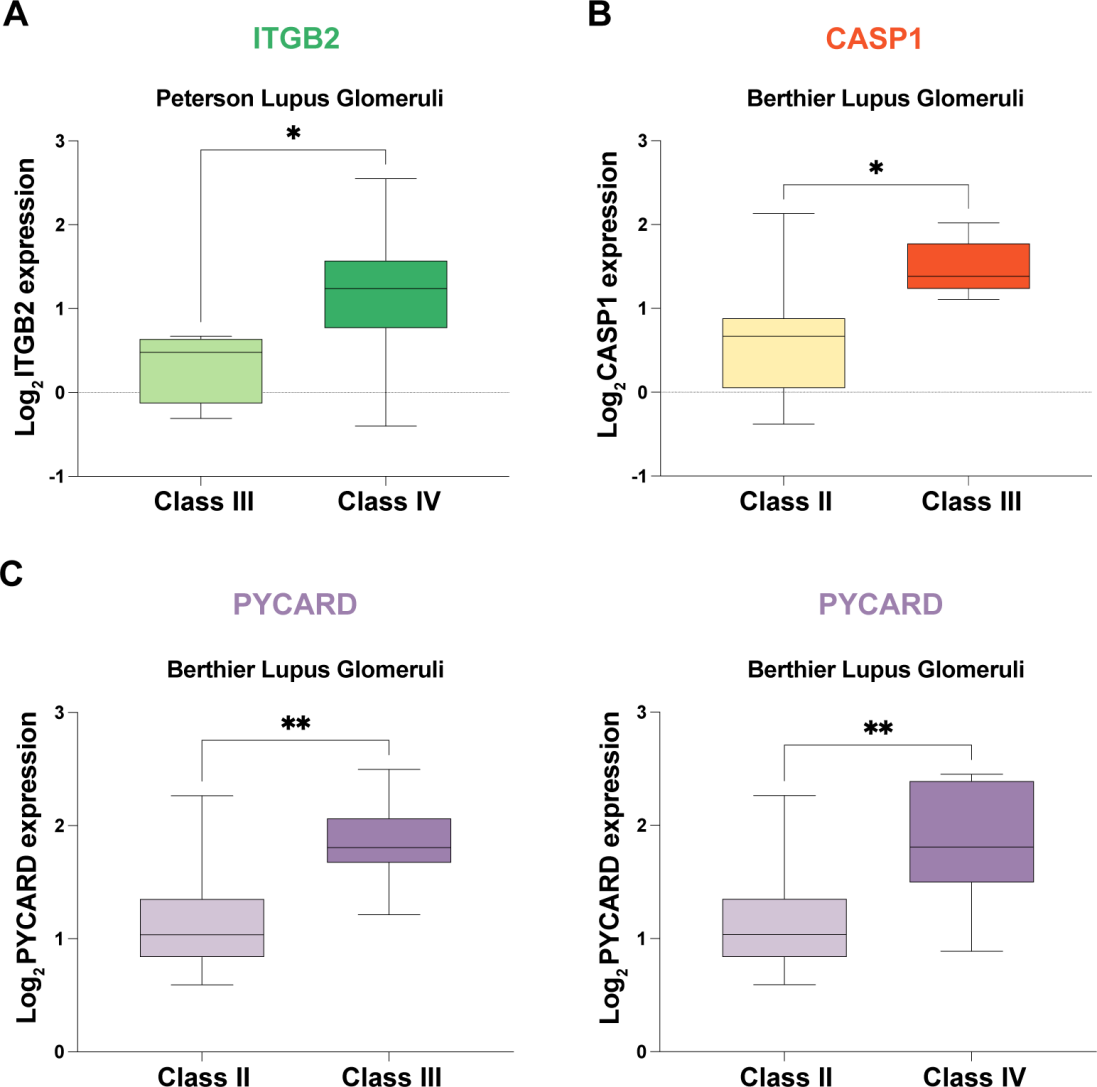
Correlation Analysis of Potential mRNA Vaccine Targets with Pathological Stage in Two External Validation Cohorts. **(A)** Increased expression of *ITGB2* in pathological stage class IV patients compared with class III patients. **(B)** Elevated *CASP1* expression in pathological stage class III patients compared with class II patients. **(C)** *PYCARD* was more highly expressed in patients with pathological stage III disease than in patients with Class II disease and in patients with Class IV disease than in patients with Class III disease. * p < 0.05, ** p < 0.01.

The expression of potential mRNA vaccine targets was correlated with pathological stage in two validation cohorts: (1) *ITGB2*: Increased expression was observed in pathological stage Class IV compared to Class III (**Figure 7A**). (2) *CASP1* expression was higher in patients with pathological stage III disease than in patients with pathological stage II disease (**Figure 7B**). (3) *PYCARD*: Elevated expression was found in pathological stage Class III compared to Class II and in Class IV compared to Class II (**Figure 7C**).

### Expression validation in in-house cohorts

**Figure 8:**
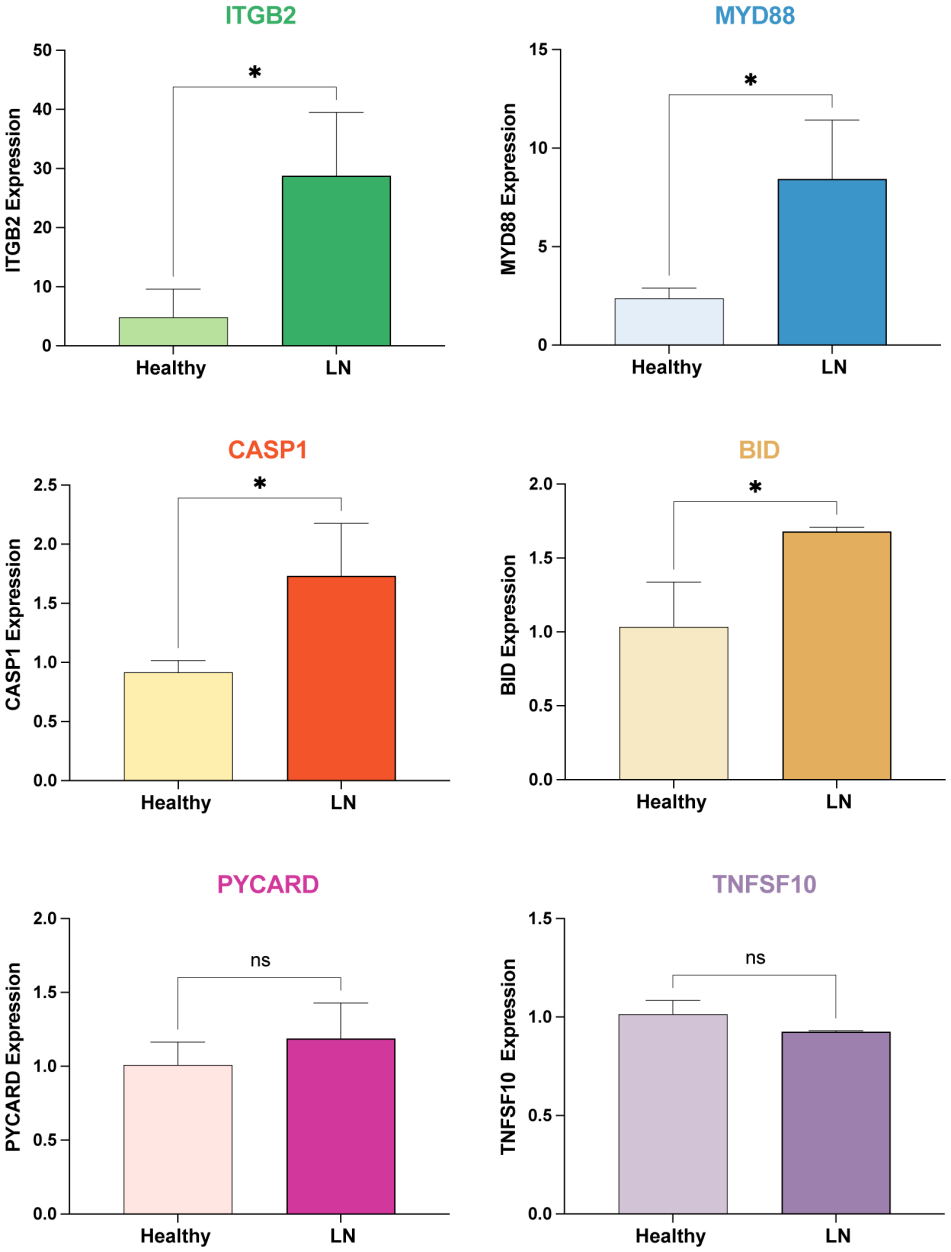
Real-time PCR Analysis of mRNA Expression Levels of Potential mRNA Vaccine Targets. The mRNA expression levels of *ITGB2, MYD88, CASP1, BID, PYCARD*, and *TNFSF10* were examined in our in-house cohorts of LN patients and healthy controls using real-time PCR analysis. * p < 0.05, ns: not significant.

The mRNA expression levels of potential mRNA vaccine targets (*ITGB2, MYD88, CASP1, BID, PYCARD*, and *TNFSF10*) were examined in our in-house cohorts of LN patients and controls using real-time PCR analysis, further validating the findings (**Figure 8**).

## Discussion

Our study represents a pivotal advancement in the field of precision medicine, particularly in the context of lupus nephritis (LN) research. By employing the technology of single-cell RNA sequencing, we have mapped the complex immunological landscape of LN in unprecedented detail. This approach has not only confirmed the findings of previous research (7, 18) but also significantly expanded our understanding, particularly in terms of the dynamic cellular processes within LN.

The implementation of non-negative matrix factorization (NMF) on single-cell data has been particularly transformative, moving beyond static snapshots of the immune environment in LN (18, 39) to reveal dynamic leukocyte meta-programs. The application of NMF allowed us to uncover four distinct leukocyte meta-programs within LN samples, each representing a unique transcriptional signature that highlights different aspects of immune function and cellular states. These findings offer a deeper understanding of LN, providing insights into the underlying mechanisms of immune dysregulation and suggesting potential targets for therapeutic intervention, particularly in the realm of mRNA vaccine development.

In this study, we selected four specific gene groups (MTOR-related genes, autophagy-related genes, Toll-like receptor-related genes, and adaptive immune system-related genes) because of their essential roles in crucial biological processes and pathways implicated in LN pathogenesis. The mTOR signaling pathway is crucial for cell growth, proliferation, and survival. Dysregulation of this pathway has been implicated in various autoimmune diseases, including LN, where it influences immune cell metabolism and function. Understanding the role of MTOR-related genes can provide insights into the metabolic aspects of LN and potential therapeutic targets. Autophagy is a fundamental cellular process involved in the degradation and recycling of cellular components. It plays a significant role in maintaining cellular homeostasis and regulating immune responses. In LN, autophagy has been linked to both protective and pathogenic effects, making it a critical area of study for understanding disease mechanisms and identifying therapeutic interventions.

**Figure 9:**
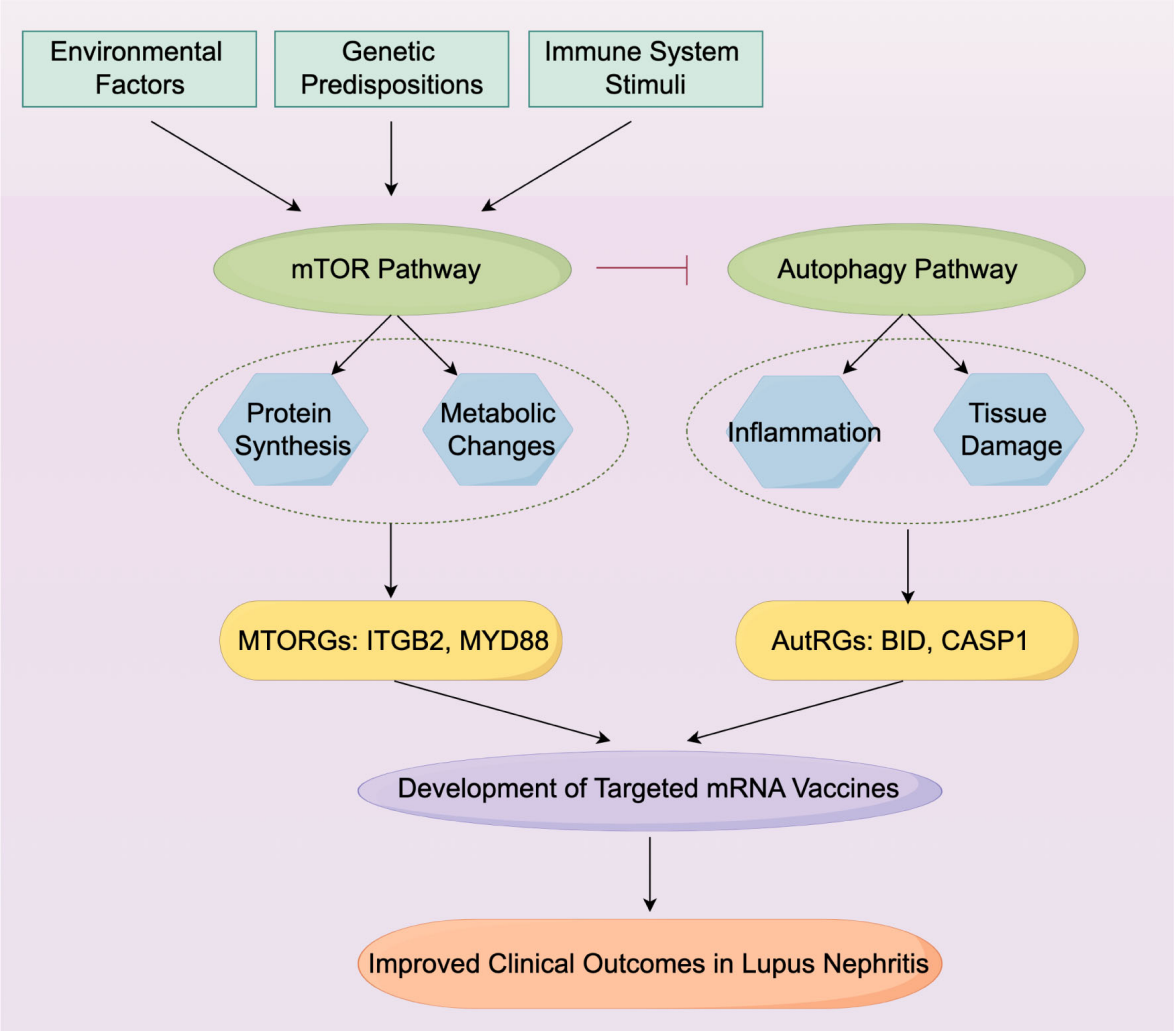
Mechanistic Diagram of mTOR Pathway Activation and Autophagy Suppression in LN. The diagram represents the molecular interactions and pathways activated by environmental and genetic triggers leading to LN. It highlights the dual role of the mTOR pathway in enhancing protein synthesis and metabolic activities while suppressing autophagy. Critical mRNA vaccine targets identified within the MTORGs and AutRGs pathways are shown, with potential implications for mRNA vaccine development aimed at modulating the immune response in LN. The targets identified, *ITGB2* and *MYD88* from the MTORGs, along with *BID* and *CASP1* from the AutRGs, each play a crucial role in the pathophysiology of LN.

In further detailing the implications of our findings, we have illustrated the complex interplay of the mTOR and autophagy pathways and their implications for mRNA vaccine development in a mechanistic diagram (**Figure 9**). This diagram summarizes the activation of the mTOR pathway by various triggers, leading to the subsequent suppression of autophagy, and identifying specific mRNA targets within these pathways—namely MTORGs (*ITGB2* and *MYD88*) and AutRGs (*BID* and *CASP1*). It shows how dysregulation of these pathways contributes to the pathogenesis of LN and underscores potential therapeutic intervention points for mRNA-based strategies. Renal mTORC1 activation, which has been associated with disease activity and prognosis in LN, is significantly activated in podocytes, mesangial cells, endothelial cells, and tubular epithelial cells (40, 41). This activation correlates strongly with clinical indicators such as serum albumin, complement C3, proteinuria, and other pathological biomarkers. Meanwhile, autophagy, generally inhibited by mTOR activation, plays a crucial role in immune system regulation, affecting T and B cell differentiation and the function of antigen-presenting cells (42–44). The intricacies of these pathways underscore the potential for therapeutic interventions targeting mTOR and autophagy to modulate disease progression in lupus nephritis. This illustration serves to bridge our comprehensive genomic analysis with practical therapeutic applications, underlining the translational potential of our study.

Toll-like receptors (TLRs) are essential components of the innate immune system and are responsible for recognizing pathogen-associated molecular patterns and initiating immune responses. Dysregulation of TLR signaling has been associated with increased inflammation and autoimmunity in patients with LN. Investigating TLR-related genes will help elucidate the contribution of innate immune responses to LN pathogenesis.

The adaptive immune system is central to the development and progression of autoimmune diseases. Genes involved in the adaptive immune response, including those regulating T and B-cell function, are critical for understanding the immunopathology of LN. Studying AISRGs provides insights into the mechanisms of immune regulation and potential targets for immunomodulatory therapies.

We developed 417 predictive models using 12 machine learning algorithms, focusing on key gene sets related to mTOR, autophagy, Toll-like receptors, and adaptive immune system signaling pathways. The high predictive accuracy of these models, particularly those utilizing combinations of glmBoost and naive Bayes algorithms for MTORGs (mean AUC=0.927) and Stepglm [both] and naive Bayes for AutRGs (mean AUC=0.903), underscores their potential in identifying key molecular targets for LN treatment. These results suggest that certain gene interactions and expression patterns are pivotal in LN pathogenesis and could be exploited for therapeutic purposes.

The exceptional predictive strength of models focusing on MTORGs and AutRGs indicates that these pathways play critical roles in LN. By targeting these pathways, we can potentially develop mRNA vaccines that modulate specific immune responses. The identification of key genes within these pathways provides a foundation for designing personalized mRNA vaccines aimed at correcting immune dysregulation in LN patients.

In the area of predictive analytics, our study underscores the potential of computational biology with an extensive array of machine learning algorithms. Our suite of 417 predictive models transcends conventional approaches, encapsulating the subtleties of disease progression and patient heterogeneity. This rich predictive framework not only aids in refining diagnostic techniques but also plays a critical role in identifying potential mRNA vaccine targets.

Our validation of potential mRNA vaccine targets included analyzing their expression levels using five external datasets and correlating them with clinical parameters such as the glomerular filtration rate (GFR) and pathological stage. These analyses confirmed the clinical relevance of these targets, highlighting their potential roles in LN pathophysiology and as targets for mRNA vaccine development. Furthermore, we validated the mRNA expression levels of these targets within our in-house cohorts using quantitative real-time PCR. This step was crucial for verifying the consistency of our findings across different cohorts and experimental conditions, thereby reinforcing the robustness of our identified targets.

The integration of functional network analysis using GeneMANIA has provided valuable insights into the roles of central hub genes in LN. This network-oriented view has allowed us to gain a holistic understanding of the molecular interactions of LN, which is crucial for identifying key antigenic targets for mRNA vaccine development and offering a path toward more personalized and effective treatments. By mapping these complex interactions, we identified potential molecular targets central to LN pathophysiology, enhancing our understanding of the underlying disease mechanisms and highlighting novel therapeutic targets.

The synergistic use of single-cell genomics, NMF, and machine learning in our research shed light on LN treatment. We not only elucidated the mechanisms of the disease but also established a foundation for the development of targeted mRNA vaccines. These vaccines, which were designed to address the specificities of LN, could revolutionize the therapeutic landscape for this condition.

While our study provides valuable insights into potential mRNA vaccine targets for LN, several limitations must be acknowledged. Personalized mRNA vaccines rely heavily on patient-specific genomic information, protein profiles, and gene expression data. Our current results, although promising, are limited in their capacity to fully capture the personalized nature required for effective mRNA vaccine development.

In conclusion, our study has significantly enriched LN research by introducing novel methodologies that set new standards for the investigation of autoimmune diseases. These advancements hold immense promise for personalized patient care. In the future, the continuation of research based on our findings is expected to refine both diagnostic and therapeutic tools, facilitating in an era of improved outcomes for patients with LN, particularly through the development of custom-designed mRNA vaccines.

## Data Availability

The single-cell RNA sequencing data were accessed from the ImmPort database (accession code SDY997: https://www.immport.org/shared/study/SDY997). The bulk RNA sequencing datasets used were obtained from the GSE32591 (https://www.ncbi.nlm.nih.gov/geo/query/acc.cgi?acc=GSE32591), GSE113342 (https://www.ncbi.nlm.nih.gov/geo/query/acc.cgi?acc=GSE113342), GSE200306(https://www.ncbi.nlm.nih.gov/geo/query/acc.cgi?acc=GSE200306), and GSE81622(https://www.ncbi.nlm.nih.gov/geo/query/acc.cgi?acc=GSE81622).

## Supplementary material

Supplementary Figure 1 Additional t-SNE plots detailing the distribution of specific marker genes of four major cell types, namely, **(A)** B cells, **(B)** epithelial cells, **(C)** myeloid cells, and **(D)** T/NK cells.

Supplementary Figure 2 Diagnostic precision of the predictive models in the training cohort. The C-indexes for the predictive models across MTORG, AutRG, TolRG, and AISRG in the GSE32591 and GSE113342 training cohorts stratified by gene set and algorithm combination.

Supplementary Figure 3 Validation of the efficacy of the predictive models in the renal sample cohort (Test 1 cohort). The performance of the predictive models across MTORG, AutRG, TolRG, and AISRG in the GSE200306 cohort.

Supplementary Figure 4 Predictive model validation in the blood sample cohort (Test 2 cohort). The performance outcomes across MTORG, AutRG, TolRG, and AISRG in the GSE81622 cohort delineate the diagnostic potential of the models in a noninvasive context.

Supplementary Figure 5 Single-cell expression of the hub genes. Dot plot showing the expression patterns of the **(A)** MTORG, **(B)** AutRG, **(C)** TolRG, and **(D)** AISRG hubs across various cell types within the single-cell dataset.

Supplementary Figure 6 The intersection of the functional enrichments across gene sets. The intersection of the functional enrichments across four gene sets (MTORGs, AutRGs, TolRGs, and AISRGs) was visualized with an UpSet diagram.

Supplementary Table 1 Details of the external datasets used for validation.

Supplementary Table 2 Machine learning algorithms and their parameter configurations.

Supplementary Table 3 Specific primer sequences for q-PCR analysis.

Supplementary Table 4 Details of the external datasets used for validation.

Supplementary Table 5 Gene list corresponding to **Figure 2B**.

Supplementary Table 6 MTORG Model Performance.

Supplementary Table 7 AutRG Model Performance.

Supplementary Table 8 TolRG Model Performance.

Supplementary Table 9 AISRG Model Performance.

Supplementary Table 10 The GO enrichment results for MTORGs.

Supplementary Table 11 The GO enrichment results for AutRGs.

Supplementary Table 12 The GO enrichment results for TolRGs.

Supplementary Table 13 The GO enrichment results for AISRGs.

Supplementary Table 14 The intersection of the functional enrichments across four gene sets.
