# Supplementary figures and images for: Integrating genomics and AI to uncover molecular targets for mRNA vaccine development in lupus nephritis
Source: Front Immunol. 2024 Oct 4;15:1381445. doi: 10.3389/fimmu.2024.1381445 (PMC11486652; doi:10.3389/fimmu.2024.1381445)

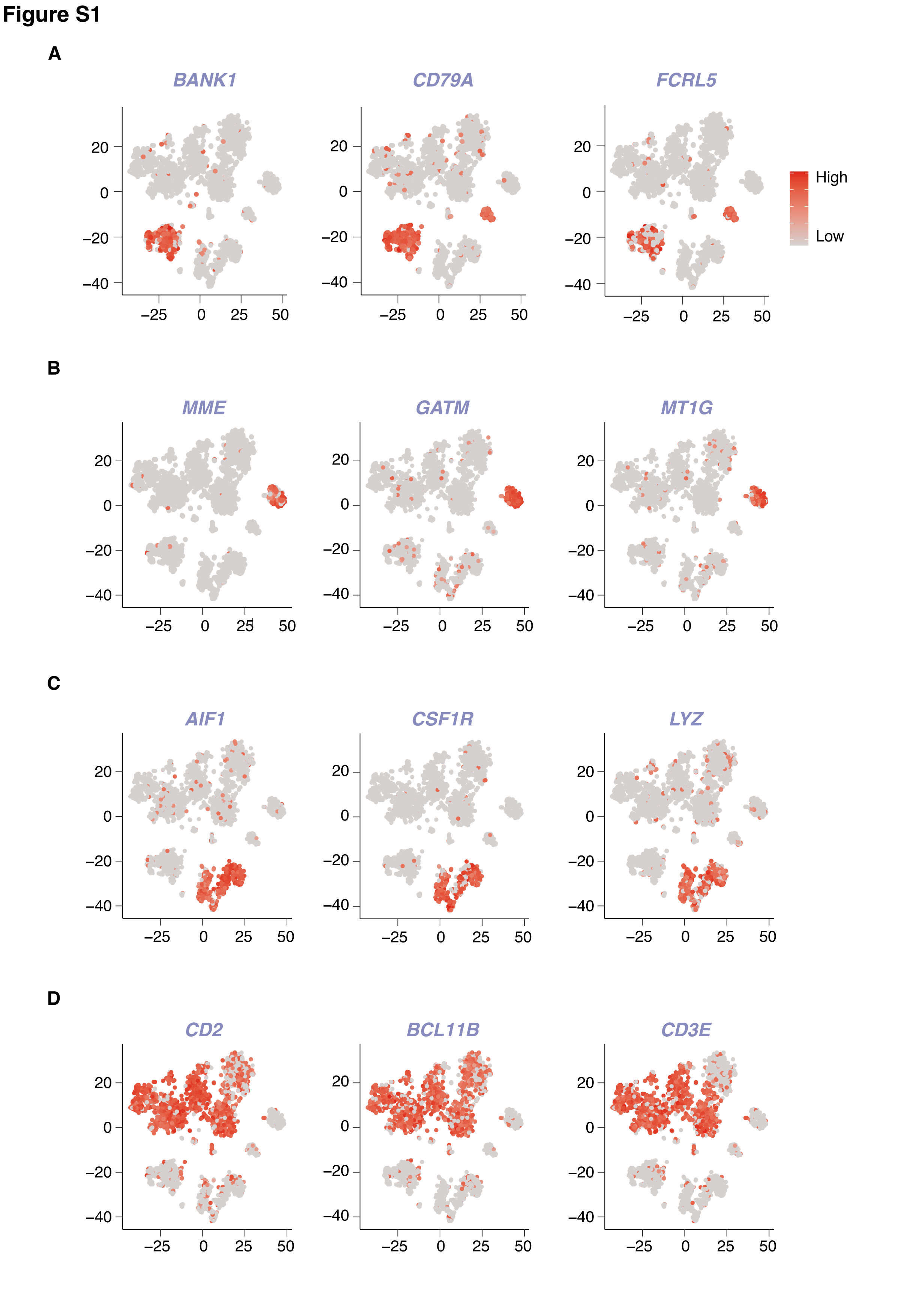

Supplement: Supplementary Figure 1 — Additional t-SNE plots detailing the distribution of specific marker genes of four major cell types, namely, (A) B cells, (B) epithelial cells, (C) myeloid cells, and (D) T/NK cells. [file Image1.tif]

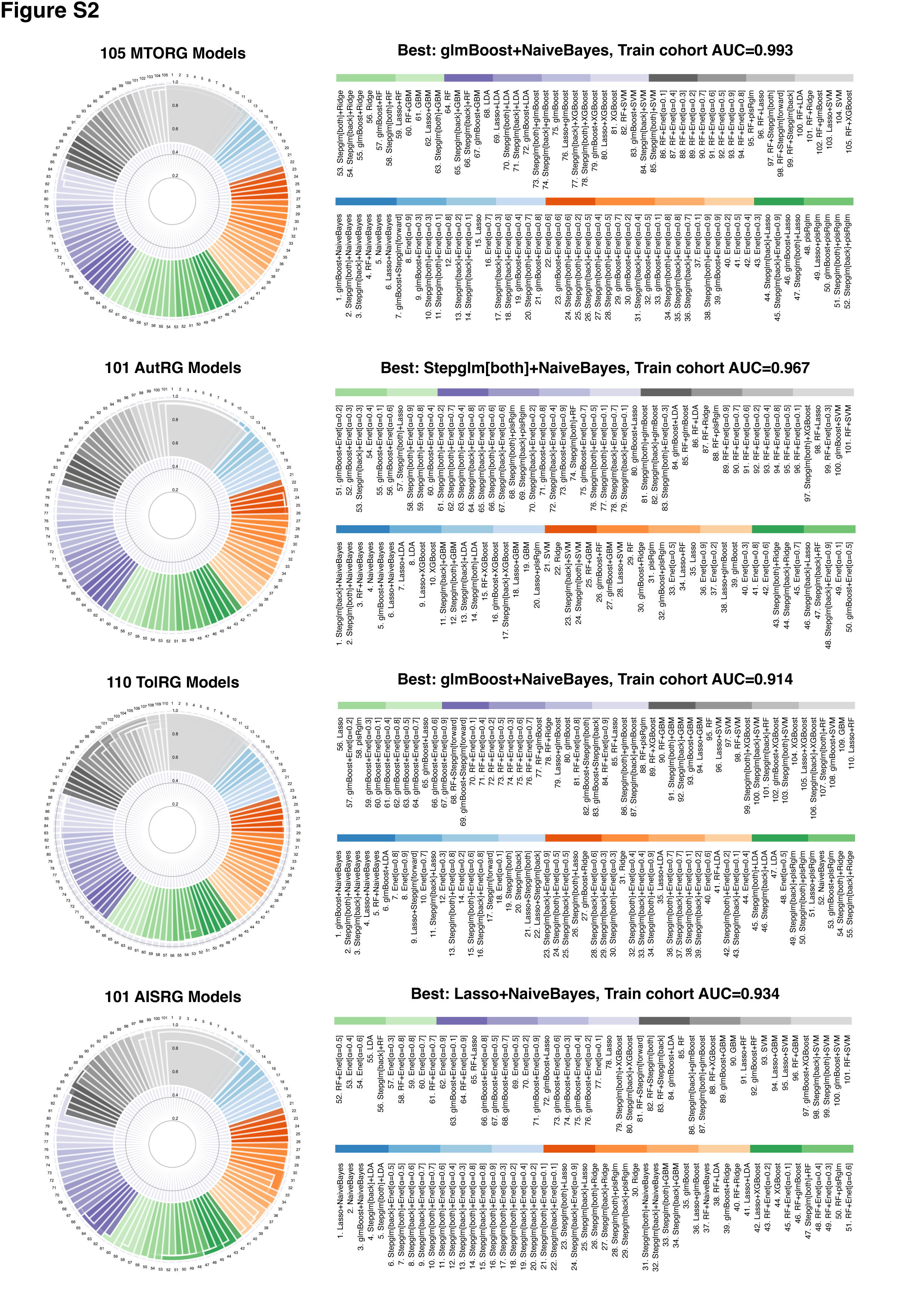

Supplement: Supplementary Figure 2 — Diagnostic precision of the predictive models in the training cohort. The C-indexes for the predictive models across MTORG, AutRG, TolRG, and AISRG in the GSE32591 and GSE113342 training cohorts stratified by gene set and algorithm combination. [file Image2.tif]

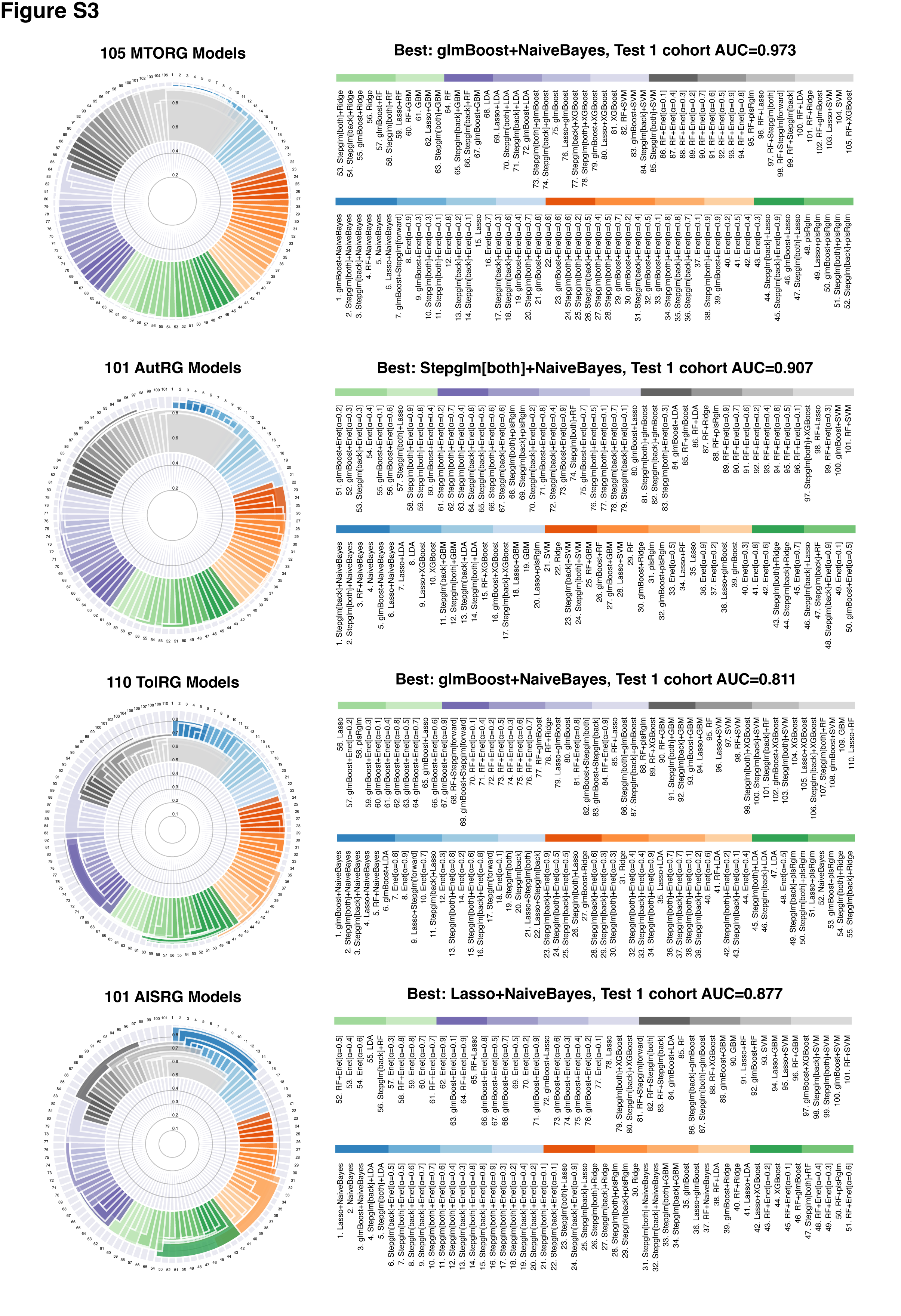

Supplement: Supplementary Figure 3 — Validation of the efficacy of the predictive models in the renal sample cohort (Test 1 cohort). The performance of the predictive models across MTORG, AutRG, TolRG, and AISRG in the GSE200306 cohort. [file Image3.tif]

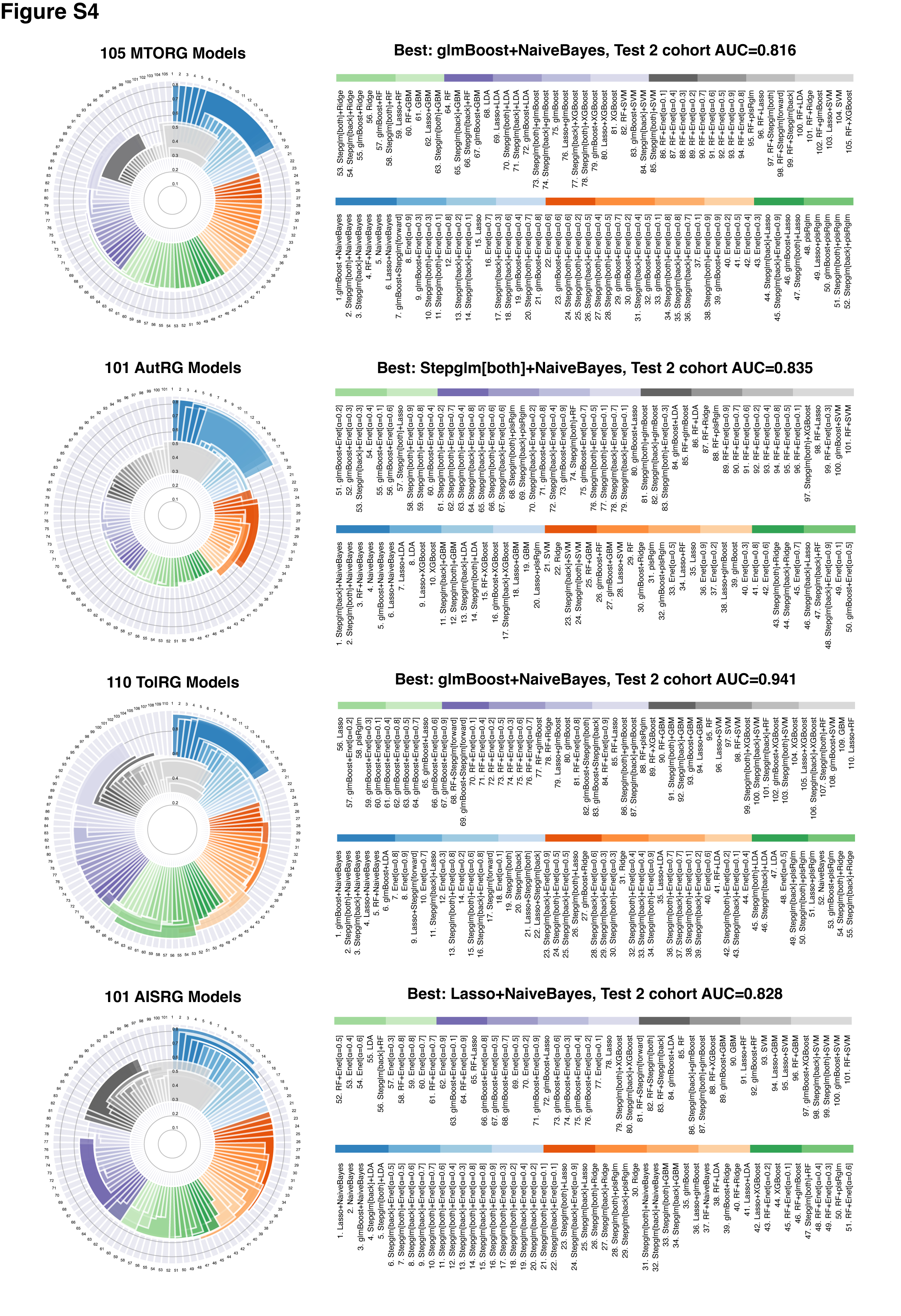

Supplement: Supplementary Figure 4 — Predictive model validation in the blood sample cohort (Test 2 cohort). The performance outcomes across MTORG, AutRG, TolRG, and AISRG in the GSE81622 cohort delineate the diagnostic potential of the models in a noninvasive context. [file Image4.tif]

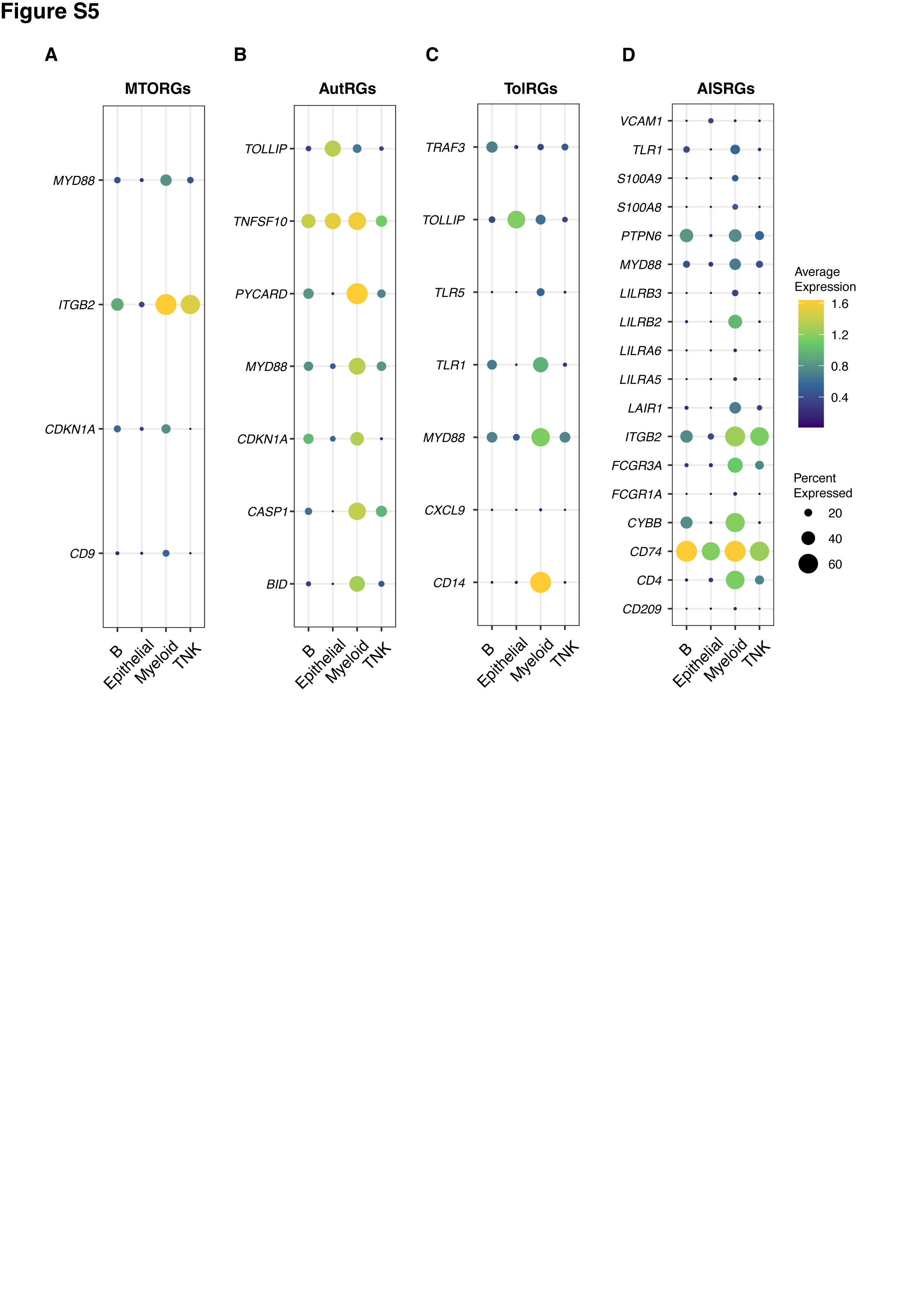

Supplement: Supplementary Figure 5 — Single-cell expression of the hub genes. Dot plot showing the expression patterns of the (A) MTORG, (B) AutRG, (C) TolRG, and (D) AISRG hubs across various cell types within the single-cell dataset. [file Image5.tif]

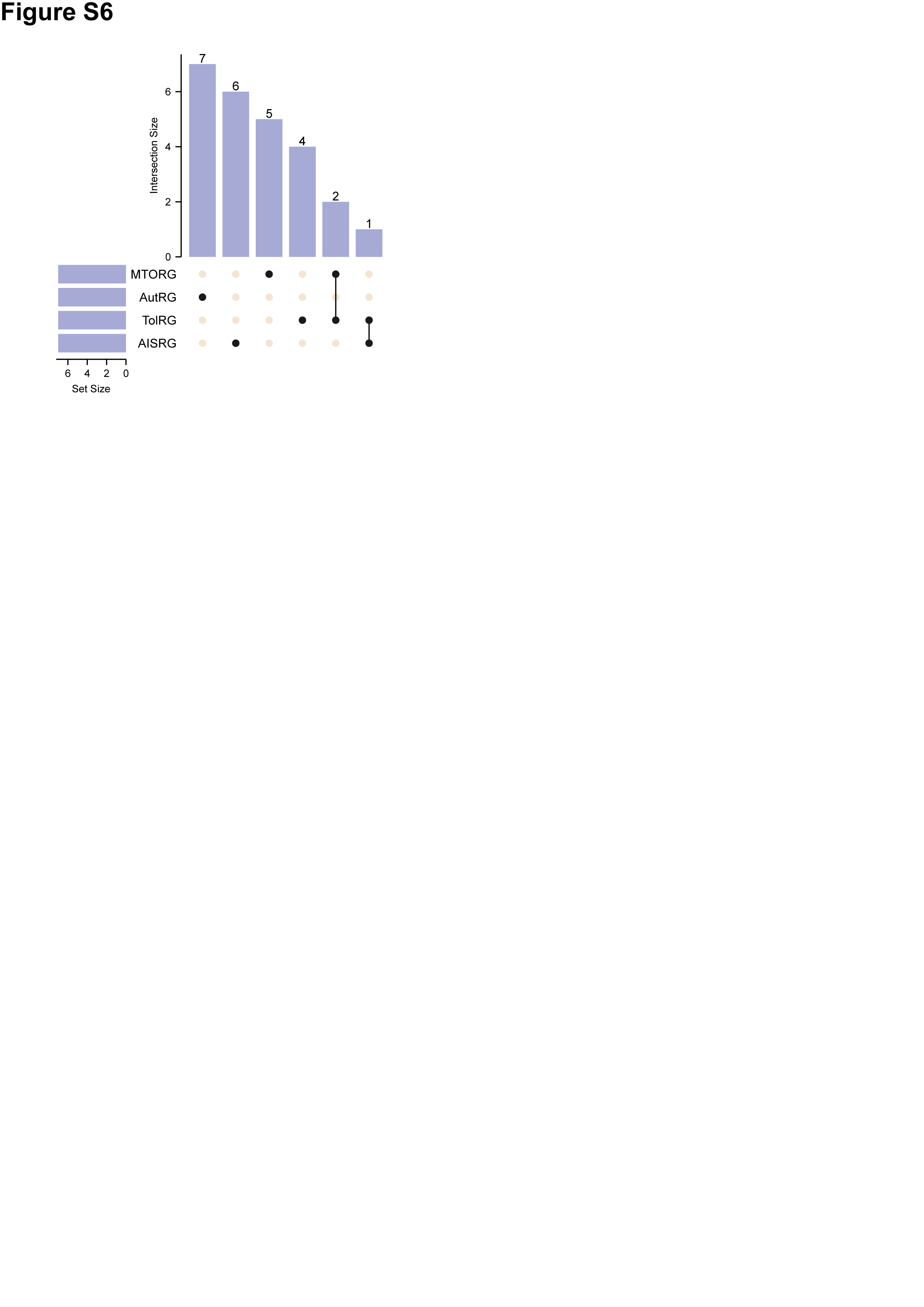

Supplement: Supplementary Figure 6 — The intersection of the functional enrichments across gene sets. The intersection of the functional enrichments across four gene sets (MTORGs, AutRGs, TolRGs, and AISRGs) was visualized with an UpSet diagram. [file Image6.tif]
